# Progesterone/Org inhibits lung adenocarcinoma cell growth via membrane progesterone receptor alpha

**DOI:** 10.1111/1759-7714.13528

**Published:** 2020-06-11

**Authors:** Jian Xiao, Xi Chen, Xiaoxiao Lu, Mingxuan Xie, Bixiu He, Shuya He, Shaojin You, Qiong Chen

**Affiliations:** ^1^ National Clinical Research Center for Geriatric Disorders Xiangya Hospital of Central South University Changsha China; ^2^ Department of Geriatrics, Respiratory Medicine Xiangya Hospital of Central South University Changsha China; ^3^ Department of Respiratory and Critical Care Medicine Xiangya Hospital of Central South University Changsha China; ^4^ Department of Biochemistry and Molecular Biology University of South China Hengyang China; ^5^ Laboratory of Cancer Experimental Therapy, Histopathology Core, Atlanta Research & Educational Foundation (151F), Atlanta VA Medical Center Emory University Decatur Georgia USA

**Keywords:** Adenocarcinoma, lung cancer, mPRα, progesterone

## Abstract

**Background:**

The aim of this study was to determine whether progesterone could inhibit the growth of lung adenocarcinoma cells via membrane progesterone receptor alpha (mPRα) and elucidate its potential mechanism. The relationship between mPRα expression and the survival prognosis of lung adenocarcinoma patients was studied.

**Methods:**

A mPRα knockdown lung adenocarcinoma cell line was constructed and treated with P4 and Org (a derivative of P4 and specific agonist of mPRα). Cell proliferation was assessed using CCK‐8 and plate colony formation assays. Protein expression was detected by western blotting. A nude mouse model of lung adenocarcinoma was established to assess the antitumor effect of P4/Org in vivo.

**Results:**

We initially determined that mPRα could promote the development of lung adenocarcinoma through the following lines of evidence. High expression of mPRα both at the mRNA and protein level was significantly associated with the poor prognosis of lung adenocarcinoma patients. The downregulation of mPRα inhibited the proliferation of lung adenocarcinoma cells. We further showed that mPRα mediates the ability of P4 to inhibit the growth of lung adenocarcinoma cells through the following lines of evidence: P4/Org inhibited the proliferation of lung adenocarcinoma cells; mPRα mediated the ability of P4/Org to inhibit lung adenocarcinoma cell proliferation; mPRα mediated the ability of P4/Org to inhibit the PKA (cAMP‐dependent protein kinase)/CREB (cAMP responsive element binding protein) and PKA/β‐catenin signaling pathways; and P4/Org inhibited the growth of a lung adenocarcinoma tumor model in vivo.

**Conclusions:**

In summary, the results of our study show that progesterone can inhibit lung adenocarcinoma cell growth via mPRα.

## Introduction

The mortality rate associated with lung cancer ranks first among all cancers.^1^ Non‐small cell lung cancer (NSCLC) accounts for 85% of lung cancer,^2^ and lung adenocarcinoma is an important pathological subtype of NSCLC. At present, there are many clinical treatments for lung adenocarcinoma, such as surgical resection, chemotherapy, radiotherapy, targeted therapy and the increasingly popular immunotherapy.^3^ However, lung adenocarcinoma often recurs after surgical resection and radiotherapy, while chemotherapy, targeted therapy and even immunotherapy often induce resistance.^4^ Thus, even if there are multiple treatment options for lung adenocarcinoma patients, the three‐year overall survival rate of patients with advanced lung adenocarcinoma is no more than 60%,^5^ while the five‐year overall survival rate is less than 15%.^6^ Therefore, it is necessary to find new potential therapeutic targets and to explore new treatment methods for lung adenocarcinoma.

Progesterone (P4) is an indispensable female hormone that requires progesterone receptor (PR) to mediate its activity. Studies have reported that PR is expressed in lung cancer tissues, and PR can mediate the ability of P4 to promote the development of lung cancer.^7^ An interesting question is whether P4 activity mediated by receptors other than PR (such as membrane progesterone receptors) also affect the development of lung cancer. Previous studies have shown that membrane progesterone receptors (mPRs) include mPRα, mPRβ, mPRγ, mPRδ and mPRε,^8^, ^9^ among which mPRα was first reported and is the most important progesterone membrane receptor.^10^, ^11^ Although the molecular weight of mPRα is much smaller than that of PR (PR has a molecular weight of approximately 100 kDa, while mPRα has a molecular weight of only 40 kDa^10^, ^11^, ^12^), it has seven transmembrane domains, with an intracellular carboxy terminus and an extracellular amino terminus. The carboxy terminus is coupled to a G protein, while the amino terminus can bind to the ligand.^11^ Therefore, P4 can also have important physiological and pathological effects through mPRα.

mPRα is expressed in different human cancer cells, such as breast cancer cells, ovarian cancer cells, astrocytoma cells and leukemia cells.^13^, ^14^, ^15^, ^16^ However, mPRα plays different regulatory roles in different cancer cells, and studies have shown that it can promote the development of breast cancer^16^, ^17^ but inhibit ovarian cancer and leukemia cells.^13^, ^14^ Previous investigations by our team have demonstrated that mPRα mediates the P4‐induced inhibition of lung adenocarcinoma cell migration and invasion^18^ and the improved sensitivity of lung adenocarcinoma to epidermal growth factor receptor‐tyrosine kinase inhibitors.^19^ Based on previous findings, the goal of our present study was to explore the potential therapeutic target of mPRα to discover novel and feasible treatment options for lung adenocarcinoma.

## Methods

### Bioinformatics analysis

The Human Pathology Atlas (HPA)^20^ is an online bioinformatics analysis platform developed by Swedish scientists. The HPA is a gene expression‐based database of RNA transcriptomics and protein translational omics data, which primarily collects cancer tissues and their corresponding clinicopathological data. The HPA uses a systematic approach to synthesize gene transcriptomics data and related clinical data from 17 major cancers that have clinically exceeded 8000 cancer patients.^20^ To verify the correlation between gene expression levels and survival time of cancer patients, the HPA constructed a total of more than 100 million Kaplan‐Meier plots.^20^ In our current study, the HPA was used to examine the relationship between the mRNA expression of mPRα and the prognosis of patients with lung adenocarcinoma.

### Tissue microarray (TMA) and immunohistochemistry (IHC)

A lung adenocarcinoma TMA was purchased from the National Engineering Center for BioChips (Shanghai, China). The TMA used in this study was HLug‐Ade150Sur‐01, which contains 75 lung adenocarcinoma tissues and relevant clinical overall survival follow‐up data of patients. We used immunohistochemistry (IHC) to detect the expression of mPRα in TMA and further analyzed the relationship between the level of mPRα expression and the survival prognosis of these patients with lung adenocarcinoma.

A primary antibody against mPRα for IHC was purchased from Bioworld Technology (Nanjing, China) (product number: BS2813; dilution: 1:300). The IHC experimental steps and staining scoring criteria performed in this study were previously described.^21^

### Cell lines and cell culture

The human lung adenocarcinoma cell line A549, human bronchial epithelial cell line HBE and human breast cancer cell line MCF‐7 were acquired from the American Type Culture Collection (ATCC, Manassas, VA, USA). The human lung adenocarcinoma cell line PC‐9 was kindly provided by Dr Yi‐Long Wu (Guangdong Lung Cancer Institute, Guangzhou, China). The cells were cultured in RPMI‐1640 medium (Corning, Manassas, VA, USA) supplemented with 10% fetal bovine serum (FBS; Thermo Fisher Scientific, Inc., Waltham, MA, USA) and 100 units/ml of penicillin and streptomycin. Cells were maintained in a humidified incubator at 37°C under an atmosphere containing 5% CO_2_.

### Quantitative real‐time PCR (qRT‐PCR)

Total RNA was extracted using TRIzol reagent (Invitrogen, Carlsbad, CA, USA), after which cDNA was synthesized using a PrimeScript First Strand cDNA Synthesis kit (Takara Biotech, Kusatsu, Shiga, Japan) following the manufacturer's instructions. The sequences of the primers synthesized by Sangon Biotech Co., Ltd. (Shanghai, China) used for qRT‐PCR were as follows: 5′‐GTTCCAGCAGCACAACGAG‐3′ (forward) and 5′‐AGGTGAAAGAGGCAAGGACA‐3′ (reverse) for mPRα; and 5′‐AGGGGCCGGACTCGTCATACT‐3′ (forward) and 5′‐GGCGGCACCACCATGTACCCT‐3′ (reverse) for β‐actin. mPRα mRNA expression was normalized to that of β‐actin. qRT‐PCR was performed using an Applied Biosystems 7500 Real‐Time PCR Systems instrument (Applied Biosystems, Foster City, CA, USA) using the following thermocycling conditions: an incubation at 95°C for 3 minutes followed by 40 cycles of 95°C/15 seconds and 60°C/30 seconds. Each sample was assayed in triplicate.

### Western blotting (WB)

WB was performed as described in our previous study.^22^ A primary antibody against mPRα was purchased from Bioworld Technology (product number: BS2813; dilution: 1:500). A primary antibody against Ki67 was purchased from Santa Cruz Biotechnology (product number: SC‐15402; dilution: 1:500). Primary antibodies against p‐NF‐κB, p‐β‐catenin, p‐PKA and PKA were purchased from Cell Signaling Technology (product numbers: #3033, #4176, #5661 and #5842; dilution: 1:1000, 1:1000, 1:400 and 1:2000, respectively). Primary antibodies against CREB, p‐CREB and β‐catenin were purchased from Abcam (product numbers: ab32515, ab32096 and ab32572; dilution: 1:1000, 1:5000 and 1:5000, respectively). β‐tubulin and β‐actin were used as protein loading controls, primary antibodies against which were purchased from Affinity Biosciences (product number: T0023; dilution: 1:3000) and Proteintech Group (product number: 60008‐1‐Ig; dilution: 1:5000), respectively.

### Lentiviral vector construction and transfection

The lentiviral vector used in this study to knock down the expression of PAQR7 (the gene that encodes mPRα) was constructed and packaged by GeneChem (GeneChem Co. Ltd., Shanghai, China). We utilized six recombinant lentiviral vectors for PAQR7 knockdown screening, detailed information for which is described in the Supporting Information (Fig S1 and Table S1).

Before transduction, A549 and PC‐9 cells were grown exponentially to no more than 70% confluence. Then, the medium was replaced with new medium containing 8 μg/ml polybrene, after which lentiviral vectors were added to the appropriate wells at multiplicities of infection (MOI) of 50, 100 or 200. After incubating for 18 hours, the culture supernatant was discarded, and the uninfected cells were washed. Subsequently, the cells were cultured in fresh medium for 48 hours before being observed by fluoroscopy (Figs S2‐S3).

### Drug intervention

In this study, we used two drugs: progesterone (P4) and Org (a derivative of P4, 19‐CH2P4, Org OD 02–0, abbreviated as Org). P4 was purchased from Selleck Chemicals (Houston, TX, USA), while Org was purchased from Axon Medchem (Hanzeplein 1H, Groningen, The Netherlands). Before being administered to cells, the drugs were dissolved in a small amount of DMSO and then diluted with complete cell culture medium before being serially diluted to concentrations of 10, 20, 40, 80, and 160 μM according to the concentration required for the experiments.

### Cell counting kit‐8 (CCK‐8) assay

Cell proliferation was assessed by a CCK‐8 (Beyotime, Shanghai, China) assay following the manufacturer's instructions. Cells (2 × 10^3^ cells/well) were seeded in 96‐well plates (Corning Costar) for 24 hours before the medium was changed to normal medium. Then, the cells were cultured for 24, 48 or 72 hours before 10 μL of CCK‐8 solution was added to each well and the cells were incubated at 37°C for 1 hour. Subsequently, a microplate reader (Bio‐Rad, Hercules, CA, USA) was used to detect the absorbance at 450 nm.

### Colony formation assay

A total of 200 cells per well were seeded in six‐well plates (Corning Costar) and cultured for two to three weeks in complete medium, which was refreshed every two days. Then, the cells were fixed with 4% paraformaldehyde for 30 minutes and stained with Giemsa for 15 minutes. Colonies were counted only if they contained at least 50 cells. The colony formation rate was calculated as “(colony number/seeded cell number) × 100%”.

### Immunofluorescence

Cells were seeded onto glass coverslips and cultured for 24 hours. Fixation was routinely performed by incubating cells with 4% paraformaldehyde for 20 minutes. The cells were then incubated with the primary antibody against mPRα (Bioworld, product number: BS2813, dilution: 1:200) overnight at 4°C, which was followed by an incubation with a fluorochrome‐conjugated goat anti‐rabbit secondary antibody for 1 hour at room temperature in dark. After using 1 μg/mL 4,6‐diamidino‐2‐phenylindole (DAPI) to stain cell nuclei, the slides were examined by fluorescence microscopy.

### Xenograft tumor model

This study was approved by the ethics committee of Xiangya Hospital, Central South University. A xenograft tumor model was generated as described previously^23^ with appropriate adjustments. Female BALB/c nude mice (four weeks old, 16–18 g) were purchased from Silaikejingda Experimental Animal Co. Ltd. (Changsha, China). A total of 2 × 10^6^ A549 cells were subcutaneously injected into the right armpits of the nude mice. When size of the tumors was measurable, the mice were randomly divided into three groups (control, P4 and Org). For the P4 and Org groups, the mice were intraperitoneally injected with P4 and Org at a dosage of 15 mg/kg/day for 28 days, while mice in the control group were intraperitoneally injected with an equal volume of saline. The length dimension (L) and width dimension (W) of the tumors was determined using a caliper every three days, and tumor volumes were calculated using the formula LW^2^/2. The diet, mental state, mobility, bowel movements and bodyweight of the mice was observed every two days. After four weeks, the mice were sacrificed by cervical dislocation, and their tumors, livers and kidneys were removed. The liver and kidney were subjected to hematoxylin and eosin (HE) staining to observe tissue and cell morphology.

### Statistical analysis

GraphPad Prism 6.01 (GraphPad Software, La Jolla, CA, USA) was used for statistical analysis. The measurement results are presented as the means ± standard deviation. When the data were normally distributed, the difference between two groups was analyzed by Student's *t*‐test (two‐tailed). Survival analysis results between different groups were evaluated using the Log‐rank test and presented as Kaplan‐Meier curves. Differences were considered significant when *P* < 0.05.

## Results

### High mRNA expression of mPRα is associated with poor prognosis in patients with lung adenocarcinoma

**Figure 1:**
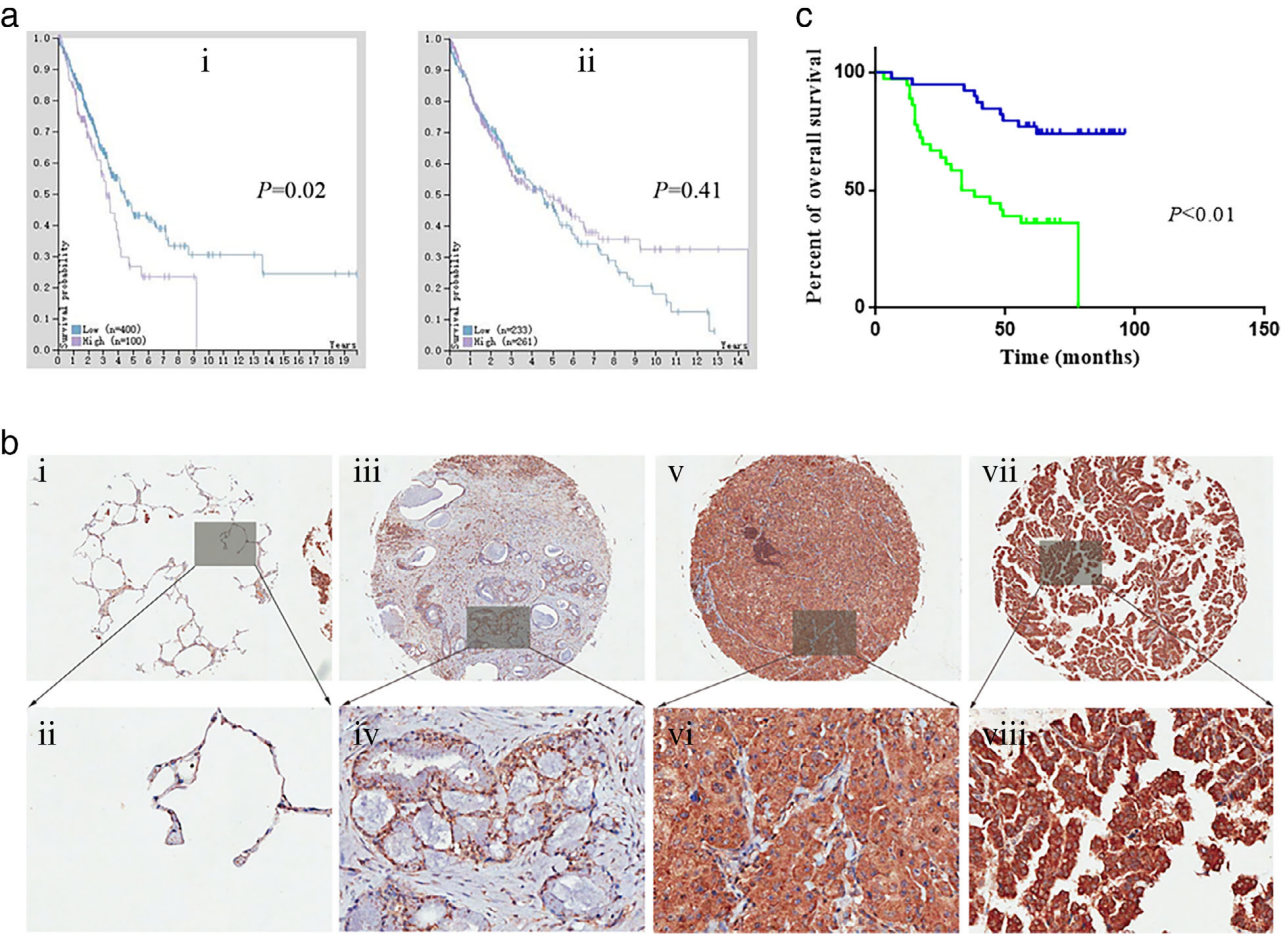
High mPRα expression is associated with poor prognosis in patients with lung adenocarcinoma. (**a**) The relationship between mPRα mRNA expression and prognosis in patients with lung adenocarcinoma (**ai**) and lung squamous cell carcinoma (**aii**) was analyzed by using HPA. (**b**) Typical images showing the IHC staining intensity of mPRα expression in different lung adenocarcinoma tissues. (**bi & bii**) Negative (paracancerous lung tissue); (**biii & biv**) weakly positive; (**bv & bvi**) moderately positive; (**bvii & bviii**) strongly positive. (**c**) Kaplan‐Meier analysis results showed that high expression of mPRα protein in lung adenocarcinoma patients (*n* = 75) is associated with poor overall survival prognosis (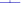) Low expression, (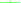) High expression. mPRα, membrane progesterone receptor alpha; HPA, Human Pathology Atlas; IHC, immunohistochemistry.

We used the HPA, an online comprehensive bioinformatics analysis platform, to study the relationship between mPRα mRNA expression level (the corresponding gene name is PAQR7) and the survival prognosis of patients with lung adenocarcinoma. The results of the analysis showed that patients with lung adenocarcinoma with high mPRα mRNA expression in cancer tissues had poor prognosis, suggesting that high mPRα mRNA expression is associated with poor prognosis (*P* < 0.05) (Fig **1**a). However, there was no significant correlation between the expression of mPRα mRNA in lung squamous cell carcinoma and the prognosis of patients (*P* > 0.05) (Fig 1a).

### High mPRα protein expression in lung adenocarcinoma suggests poor patient prognosis

We used the IHC technique to assess the expression of mPRα in TMA, which included 75 patients with lung adenocarcinoma, and to analyze the relationship between the level of mPRα expression and the survival prognosis of these patients with lung adenocarcinoma. We observed that the expression of mPRα was significantly different between cancer tissues in different patients (Fig 1b).

The typical staining intensity of mPRα in lung adenocarcinoma (or adjacent tissues) is shown in Fig 1b. The levels of mPRα expression in 75 lung adenocarcinoma tissue samples were classified by the scoring criteria,^21^ among which 39 exhibited low mPRα expression, while the other 36 tissue samples were classified in “high expression” group. When assessed together with their clinical survival follow‐up data, the results showed that patients with high mPRα expression in lung adenocarcinoma had poor overall survival prognosis, suggesting high mPRα expression is correlated with poor prognosis in patients with lung adenocarcinoma (*P* < 0.01) (Fig 1c).

### mPRα is expressed in A549 and PC‐9 lung adenocarcinoma cells and localizes to cell membrane

**Figure 2:**
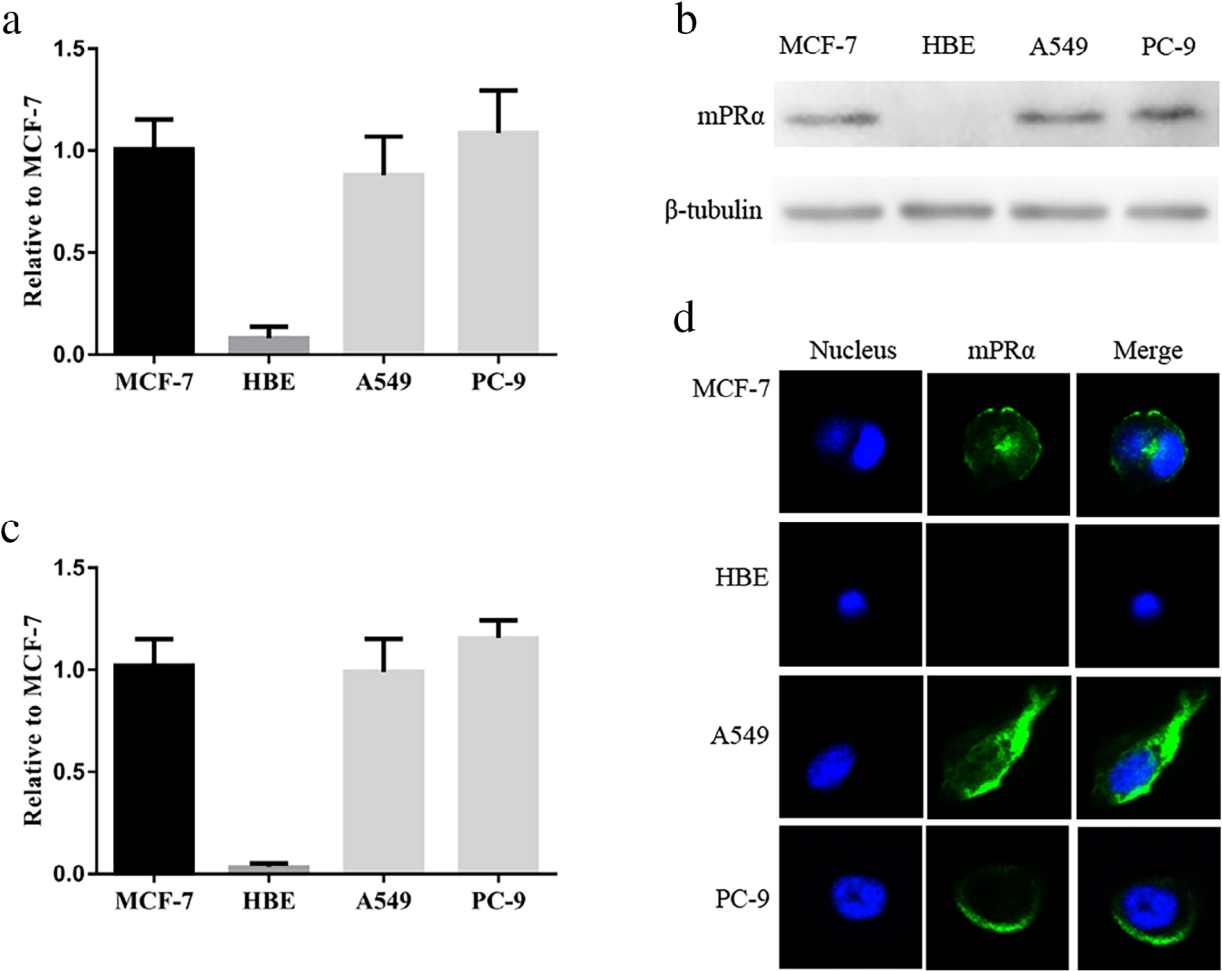
mPRα is expressed in A549 and PC‐9 lung adenocarcinoma cells and is located at the cell membrane. (**a**) mPRα mRNA expression was assessed by qRT‐PCR (*n* = 3). (**b**) The expression of mPRα protein in lung adenocarcinoma cells was detected by WB. (**c**) The gray value statistics of the WB results (*n* = 3). (**d**) Immunofluorescence of mPRα expression showed that mPRα was localized to the cell membrane. MCF‐7 was the positive control and HBE was the negative control. qRT‐PCR, quantitative real‐time PCR; WB, western blotting.

Previous studies have shown that mPRα is expressed in breast cancer MCF‐7 cells.^13^, ^17^ We used MCF‐7 and HBE cells as positive and negative controls, respectively, to assess the expression of mPRα in A549 and PC‐9 lung adenocarcinoma cells by qRT‐PCR and WB. The results showed that mPRα was significantly expressed at both the mRNA level (Fig 2a) and protein level (Fig 2b‐c), and cellular immunofluorescence results showed that mPRα localizes to the cell membrane (Fig 2d).

### Downregulation of mPRα expression can inhibit proliferation of lung adenocarcinoma cells

**Figure 3:**
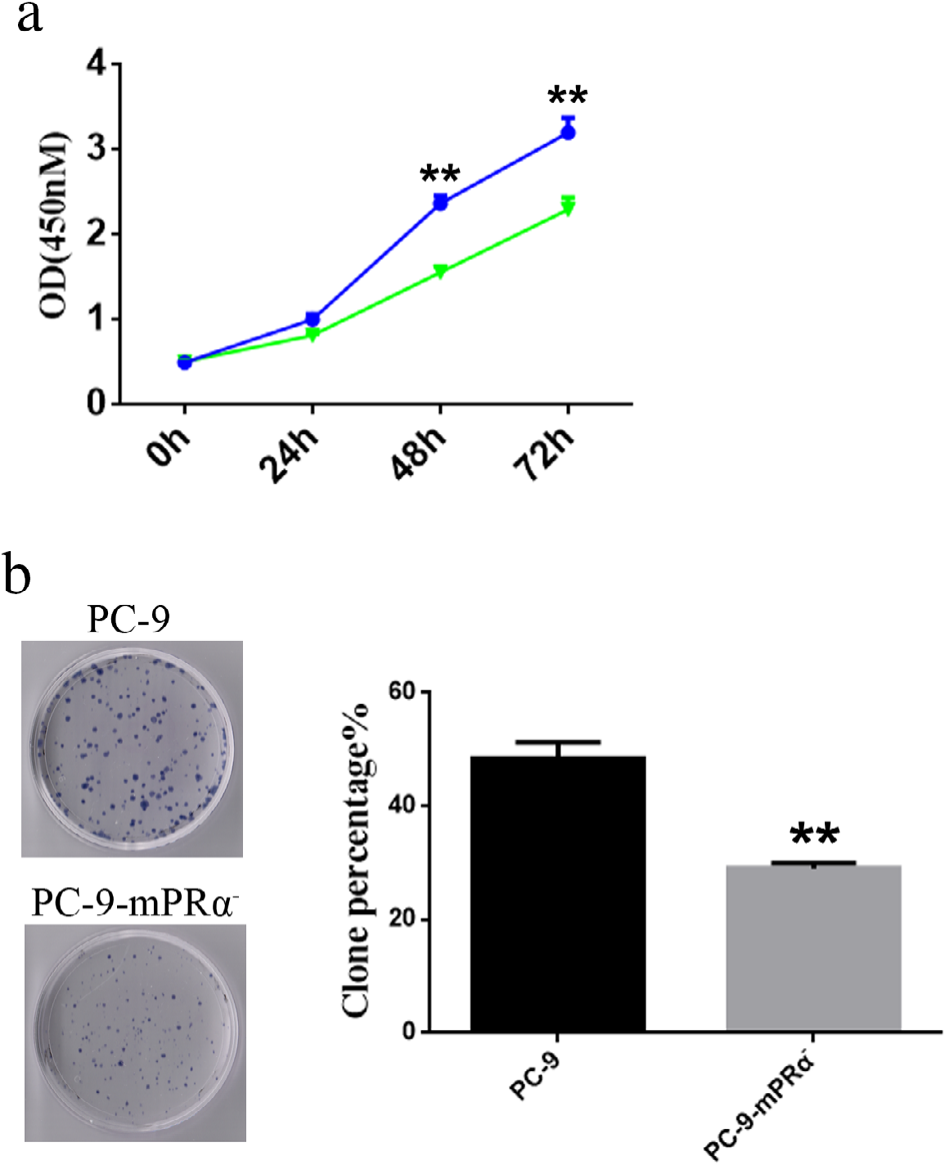
Downregulation of mPRα inhibits the proliferation of lung adenocarcinoma cells. (**a**) Cell viability was detected by a CCK‐8 assay (*n* = 3) (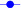) PC‐9, (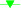) PC‐9‐mPRα. (**b**) Cell proliferation was measured by a colony formation assays (*n* = 3). CCK‐8, cell counting kit‐8. ** *P* < 0.01.

The results of the bioinformatics analysis at the mRNA level and the lung adenocarcinoma TMA at the protein level suggested that mPRα may be an important factor in promoting the growth of lung adenocarcinoma cells. Therefore, inhibition/downregulation of mPRα expression may inhibit the growth of lung adenocarcinoma cells. We used six lentiviral vectors (Lv‐44 607, Lv‐44 608, Lv‐44 609, Lv‐54 304, Lv‐54 305 and Lv‐54 306) to knockdown mPRα expression in lung adenocarcinoma cell lines (Fig S3). We observed that the lentiviral vector Lv‐44 607 exhibited a good mPRα knockdown effect (Figs S4–S5). Therefore, we performed cell function tests with lung adenocarcinoma PC‐9 cells infected with Lv‐44 607 (which we named PC‐9‐mPRα^−^ cells). The CCK‐8 and colony formation results showed that the viability and proliferation of PC‐9‐mPRα^−^ cells was significantly lower than that observed in PC‐9 cells (*P* < 0.01) (Fig. 3).

### P4/Org has an inhibitory effect on the proliferation of lung adenocarcinoma cells

The above results showed that mPRα can promote the development of lung adenocarcinoma. In a previous study, our research team showed that mPRα can mediate the ability of P4 to inhibit the migration and invasion of lung adenocarcinoma A549 cells.^18^ Therefore, we speculated that mPRα may also mediate the P4‐induced inhibition of lung adenocarcinoma cell migration to some extent.

**Figure 4:**
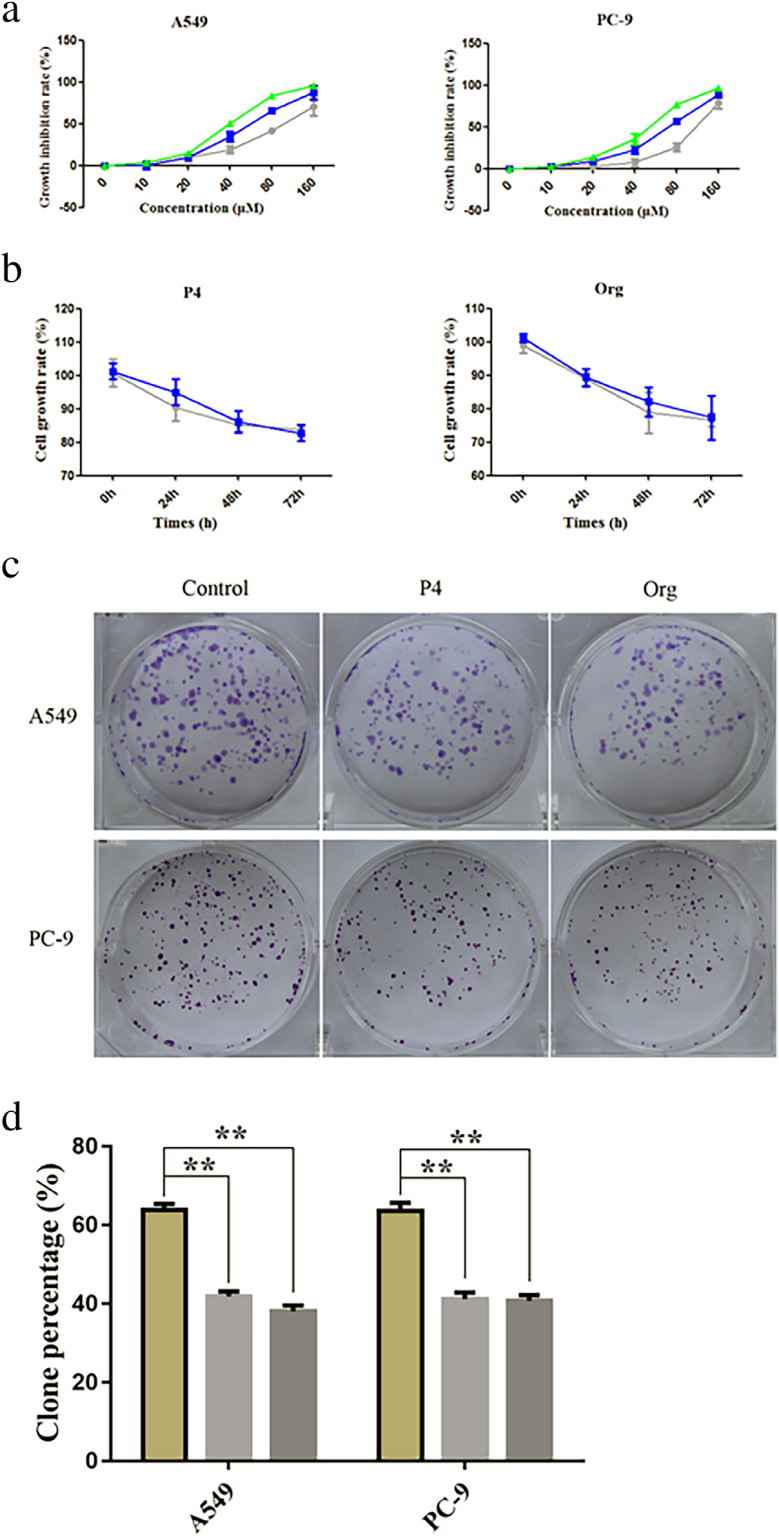
P4/Org inhibits the proliferation of lung adenocarcinoma cells. (**a**) The inhibitory effects of different concentrations of P4 (0, 10, 20, 40, 80, and 160 μM) on the growth activity of A549/PC‐9 lung adenocarcinoma cells at different times (24, 48, and 72 hours) (*n* = 3) (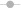) 24 hours, (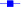) 48 hours, (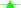), 72 hours; (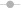) 24 hours, (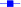) 48 hours, (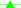), 72 hours. (**b**) The growth inhibition curves of A549/PC‐9 cells after treatment with 20 μM P4/Org (*n* = 3) (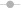) A549, (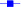) PC‐9; (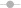) A549, (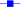) PC‐9. (**c**) Typical plate colony formation images for the P4/Org (20 μM) treatment and control groups for lung adenocarcinoma A549/PC‐9 cells. (**d**) Statistical analysis results of the colony formation assay (*n* = 3) (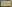) Control, (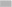) P4, (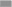) Org. ** *P* < 0.01.

We used different concentrations of P4 (0, 10, 20, 40, 80, and 160 μM) to treat lung adenocarcinoma A549 cells and PC‐9 cells for different time (24, 48, and 72 hours), and then tested cell viability by the CCK‐8 assay. The growth inhibition rate of A549 and PC‐9 cells at different concentrations of P4 was calculated. The results showed that as the P4 concentration and treatment time increased, the proliferation inhibition of A549 cells and PC‐9 cells increased gradually (Fig 4a). We further calculated the half maximal inhibitory concentration (IC50) of P4 on A549 cells and PC‐9 cells at different time. The IC50 values for P4 against A549 cells at 24, 48 and 72 hours were 96.22, 57.72 and 40.10 μM respectively. The IC50 values for P4 against PC‐9 cells at 24, 48 and 72 hours were 108.6, 69.23 and 49.41 μM respectively (Table 1).

**Table 1:** The IC50 of A549 and PC‐9 cells treated with P4 for 24, 48 and 72 hours, respectively

Treatment time (hours)	A549		PC‐9	
	IC50 (μM)	95% CI (μM)	IC50 μM)	95% CI (μM)
24	96.22	85.54–108.2	108.6	101.4–116.3
48	57.72	52.68–63.25	69.23	65.38–73.30
72	40.10	38.79–41.44	49.41	46.25–52.79

IC50, half maximal inhibitory concentration;

95% CI, 95% confidence intervals.

Based on the IC50 concentration range of P4 showing activity against A549 and PC‐9 cells at different times, we used a 20 μM P4 to treat A549 and PC‐9 cells. The results showed that the 20 μM P4 treatment produced similar growth inhibition curves for these two lung adenocarcinoma cell lines (Fig 4b). We also used a derivative of P4, Org (trade name, Org OD 02–0; chemical name, 19‐CH2P4; binds more strongly to mPRα than P4 and is a specific agonist of mPRα^24^; in this study it simplified as “Org” and was used in parallel control experiments with P4) to treat A549 and PC‐9 at a concentration of 20 μM. The results showed that the growth inhibition curves the two lung adenocarcinoma cells treated with 20 μM Org were also similar (Fig 4b). Furthermore, A549/PC‐9 cells were treated with 20 μM P4/Org in the plate formation assay. Similarly, the results showed that the 20 μM P4/Org treatment could significantly inhibit the colony formation of lung adenocarcinoma A549/PC‐9 cells (*P* < 0.01) (Fig 4c–d).

### Lung adenocarcinoma cell inhibition by P4/Org is mediated by mPRα

**Figure 5:**
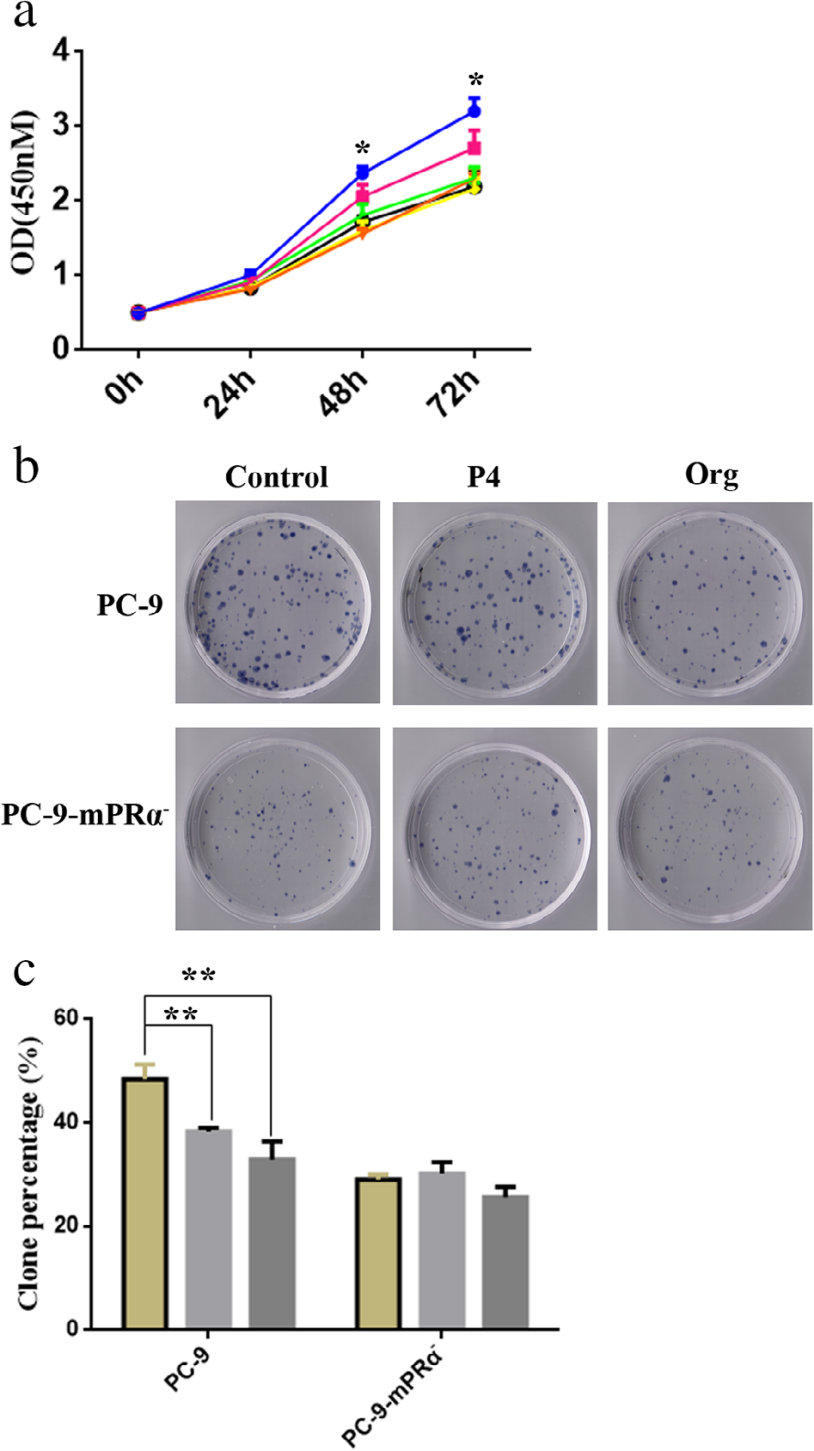
mPRα mediates the inhibitory effect of P4/Org on lung adenocarcinoma cells. (**a**) The growth curves of PC‐9/PC‐9‐mPRα^−^ cells treated with 20 μM P4/Org for different times (24, 48, and 72 hours), as detected by a CCK‐8 assay (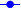) PC‐9, (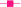) PC‐9+P4, (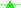) PC‐9+Org, (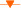) PC‐9‐mPRα^−^, (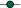) PC‐9‐mPRα^−^+P4, (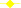) PC‐9+mPRα^−^+Org. (**b**) Effects of 20 μM P4/Org treatment on the formation of PC‐9 and PC‐9‐mPRα^−^ cell colonies. (**c**) Analysis of the results of colony formation experiments (*n* = 3) (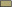) Control, (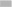) P4, (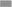) Org. * *P* < 0.05; ** *P* < 0.01.

To further elucidate whether the inhibitory effect of P4/Org toward the proliferation of lung adenocarcinoma cells was mediated by mPRα, we treated PC‐9‐mPRα^−^ and PC‐9 cells with 20 μM P4/Org for 24, 48 and 72 hours, and then evaluated them by the CCK‐8 assay. The results showed that P4/Org significantly inhibited the activity of PC‐9 cells at 48 and 72 hours (*P* < 0.05) but did not inhibit the activity of PC‐9‐mPRα^−^ cells (Fig 5a). The results of colony formation assays showed that 20 μM P4/Or had a significant inhibitory effect on the colony formation of PC‐9 cells (*P* < 0.01) but had no significant effect on that of PC‐9‐mPRα^−^ cells (Fig 5b–c). These results suggested that mPRα can also mediate the inhibitory effect of P4/Org on the proliferation of lung adenocarcinoma PC‐9 cells. Furthermore, we observed that regardless of whether they were treated with P4/Org or not, the viability and proliferative capacity of PC‐9‐mPRα^−^ cells was significantly lower than that of PC‐9 cells (Fig 5), again indicating that knockdown of mPRα expression inhibited the growth of lung adenocarcinoma PC‐9 cells.

**Figure 6:**
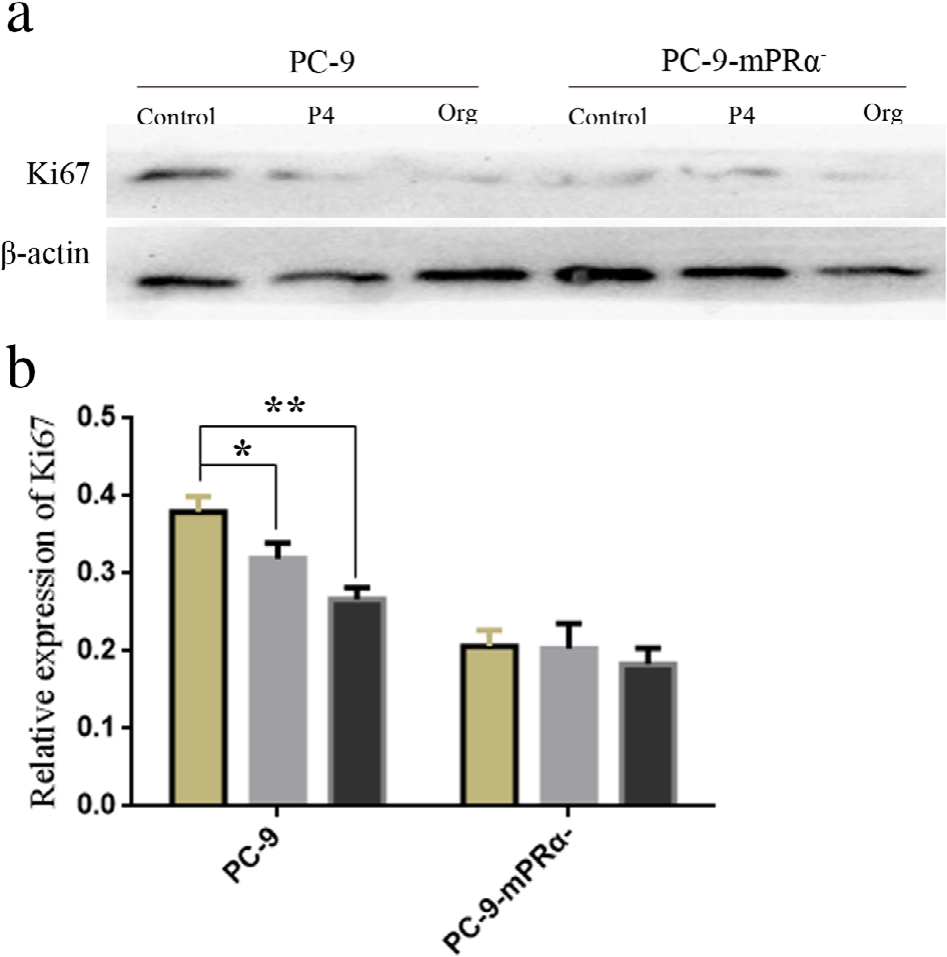
(**a**) WB detection of Ki67 expression in PC‐9 and PC‐9‐mPRα^−^ cells after 20 μM P4/Org treatment for 48 hours. (**b**) The corresponding statistical analysis results of the WB assay (*n* = 3) (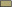) Control, (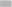) P4, (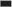) Org. *P < 0.05; **P < 0.01.

Ki67 is a well‐known biomarker of cell proliferation, as Ki67 is highly expressed in actively proliferating cells but poorly or not expressed in weakly proliferating cells.^25^ Therefore, we assessed the expression of Ki67 in PC‐9 and PC‐9‐mPRα^−^ cells treated with or without P4/Org (20 μM for 48 hours) by WB. The results showed that not only was Ki67 expression in PC‐9 cells significantly decreased after P4/Org treatment (*P* < 0.05 or 0.01), it was significantly lower in all PC‐9‐mPRα^−^ cell groups than that observed in PC‐9 cells (*P* < 0.01). At the same time, P4/Org treatment was observed to have no effect on the expression of Ki67 in PC‐9‐mPRα^−^ cells (Fig 6). These results suggested that P4/Org does inhibit the proliferation of lung adenocarcinoma cells through mPRα.

### mPRα mediates inhibition of the PKA/CREB and PKA/beta‐catenin signaling pathways by P4/Org

Previous studies have shown that P4 can mediate the production of intracellular cyclic adenosine monophosphate (cAMP) through mPRα,^26^, ^27^ and cAMP/PKA (cAMP‐dependent protein kinase) is an important pathway regulating cell growth.^28^, ^29^ Furthermore, PKA can regulate cAMP responsive element binding protein (CREB),^30^, ^31^ β‐catenin^32^, ^33^ and nuclear factor‐kappa B (NF‐κB)^34^, ^35^ by phosphorylation, allowing these proteins to play key roles in the development of cancer. Therefore, we speculated that P4/Org may influence the PKA/CREB, PKA/β‐catenin and PKA/NF‐κB pathways via mPRα.

**Figure 7:**
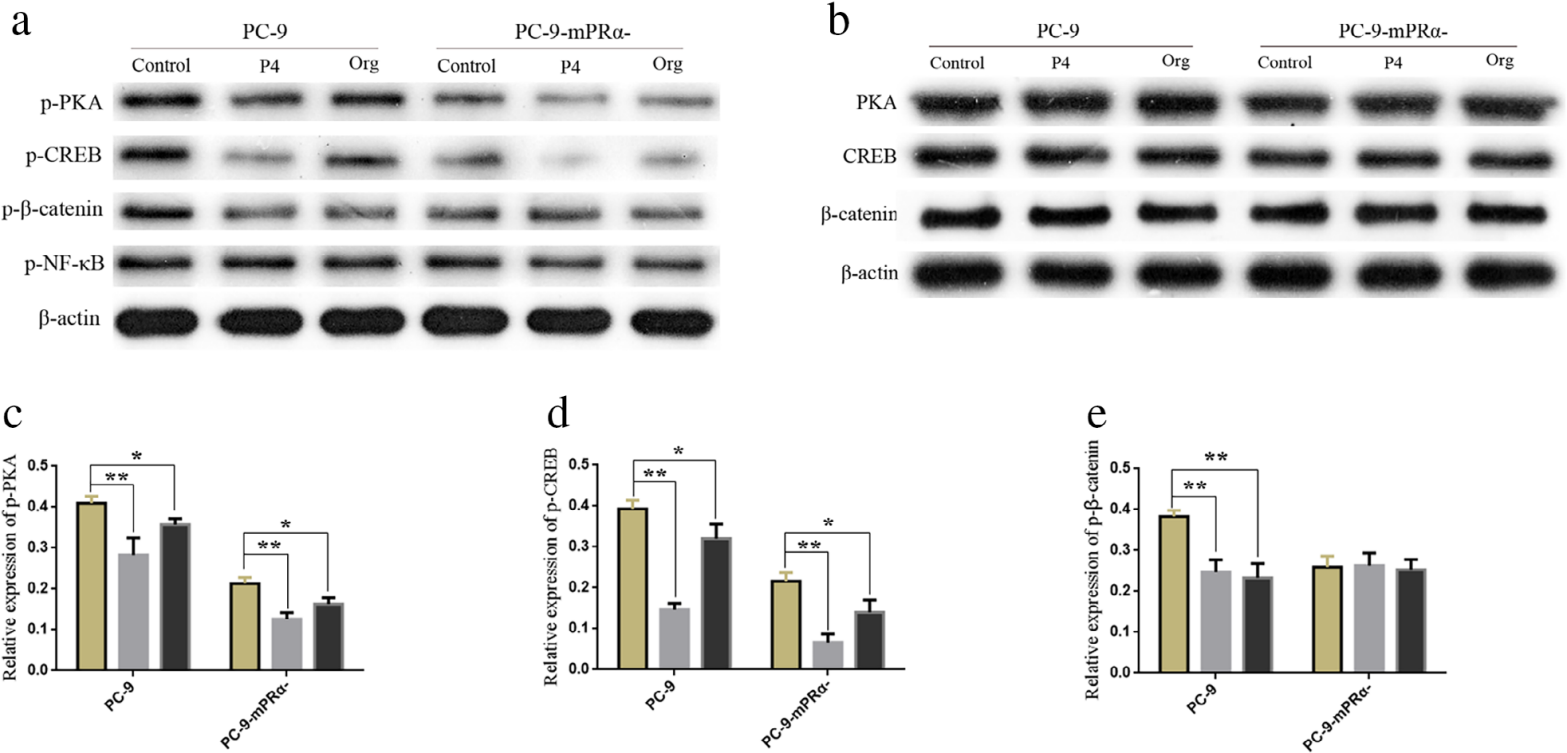
mPRα mediates the inhibitory activity of P4/Org on the PKA/CREB and PKA/beta‐catenin signaling pathways. After treating PC‐9/PC‐9‐mPRα^−^ cells with 20 μM P4/Org for 48 hours, the expression of p‐PKA, p‐CREB, p‐β‐catenin and p‐NF‐κB (**a**) and that of PKA, CREB and β‐catenin (**b**) was detected by WB in each group of cells. (**c**) The relative level of p‐PKA/β‐actin expression (*n* = 3) (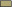) Control, (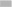) P4, (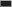) Org. (**d**) The relative level of p‐CREB/β‐actin expression (*n* = 3) (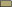) Control, (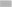) P4, (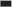) Org. (**e**) The relative level of p‐β‐catenin/β‐actin expression (*n* = 3) (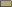) Control, (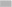) P4, (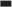) Org. *P < 0.05; **P < 0.01.

We assessed the changes in phosphorylation of proteins in the abovementioned pathways in PC‐9/PC‐9‐mPRα^−^ cells treated for 48 hours with 20 μM P4/Org. The results showed that the P4/Org treatment inhibited the phosphorylation of PKA, CREB and β‐catenin in PC‐9 cells (*P* < 0.05 or 0.01) but had no notable effect on the phosphorylation of NF‐κB (Fig 7), suggesting that mPRα can mediate P4/Org inhibition of the PKA/CREB and PKA/β‐catenin signaling pathways. Studies using PC‐9‐mPRα^−^ cells showed that P4/Org treatment also inhibited the phosphorylation of PKA and CREB but had no obvious effect on the phosphorylation of β‐catenin and NF‐κB (Fig 7). Furthermore, the P4/Org treatment did not alter the expression of total PKA, CREB and β‐catenin in PC‐9 and PC‐9‐mPRα^−^ cells (Fig 7).

### P4/Org inhibits the growth of lung adenocarcinoma in vivo

Comprehensive analysis of the in vitro results showed that P4/Org inhibited the growth of lung adenocarcinoma cells through mPRα. To determine if P4/Org could also inhibit the growth of lung adenocarcinoma in vivo, we first expanded and cultured lung adenocarcinoma A549 cells in vitro. Then, a nude mouse model of lung adenocarcinoma was generated by subcutaneously injecting the cells into the axillae of mice, which was followed by an intraperitoneal injection of P4/Org for four weeks, and the effect of the drug treatment on the growth of subcutaneous lung adenocarcinoma formation was monitored.

**Figure 8:**
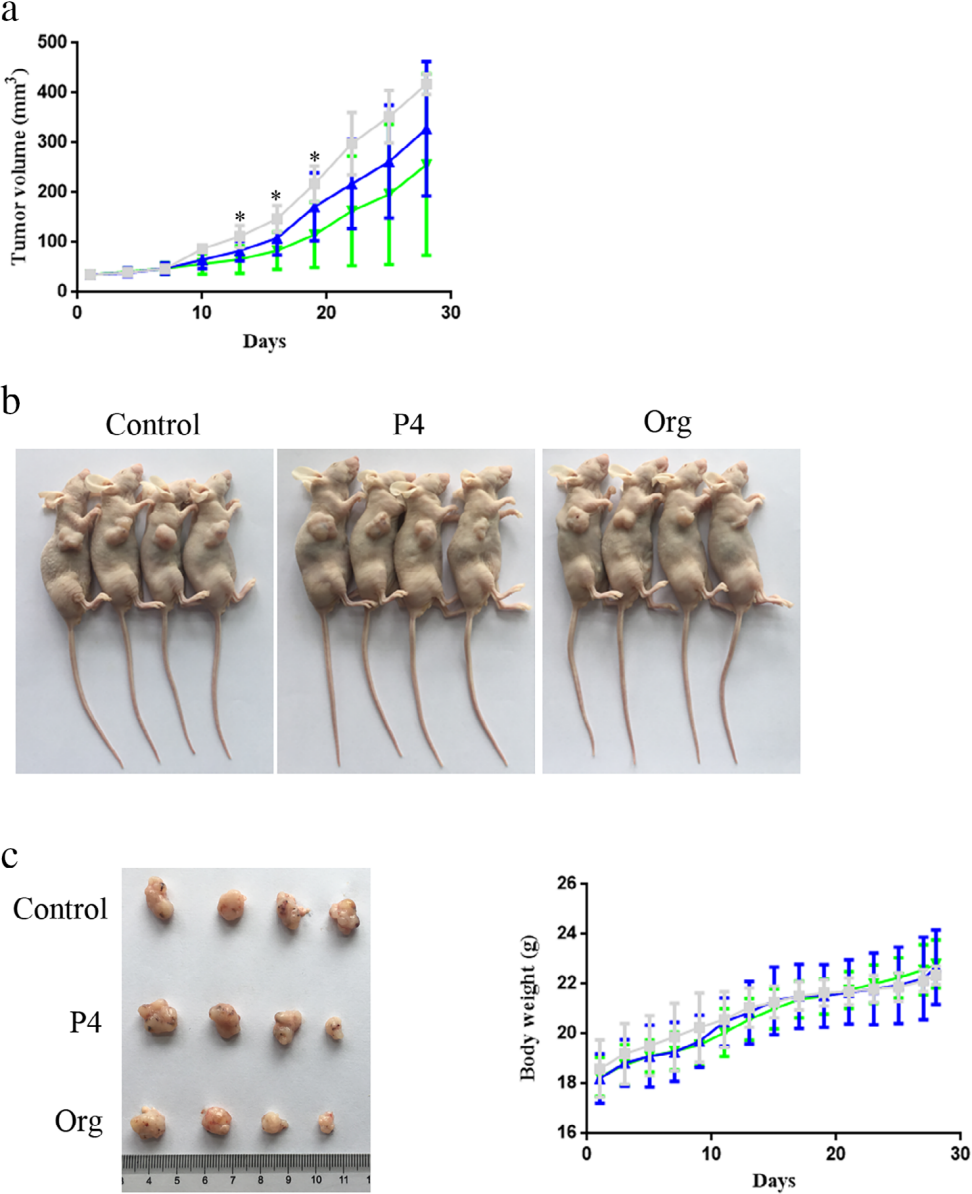
P4/Org inhibits the growth of lung adenocarcinoma in vivo. (**a**) Volume change of the subcutaneous tumor model during four weeks of P4/Org administration (15 mg/kg/day nude mouse bodyweight) (*n* = 4) (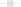) Control, (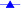) P4, (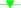) Org. (**b**) Nude mice were sacrificed after four weeks of treatment. (**c**) Comparison of the tumors from the treatment and control groups. (**d**) Changes in bodyweight of the three groups of nude mice during the four week treatment period (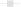) Control, (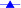) P4, (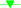) Org. *P < 0.05.

The results showed that both P4 and Org could inhibit the growth of lung adenocarcinoma models to some extent, and the anticancer effect of Org was more obvious (Fig 8a–c). Although P4 showed a tendency to inhibit the growth of lung adenocarcinoma tumor models, no significant difference was observed compared with the control group. The inhibitory effect of Org was more notable than that of P4, and it could exert a significant inhibitory effect (*P* < 0.05) in the early stage of lung adenocarcinoma tumor model growth (2–3 weeks; Fig 8a), whereas a significant difference was not observed in the later stage (after three weeks), compared to the control (Fig 8a–c). P4/Org had no effects on the diet, mental state, mobility, bowel movements and bodyweight of the nude mice after the intraperitoneal injection (Fig 8d). HE staining of liver and kidney tissues showed that the P4 and Org treatment did not cause damage to the liver and kidney of nude mice (Fig S6), indicating that P4 and Org had no obvious toxic side effects on experimental animals.

## Discussion

### mPRα promotes the development of lung adenocarcinoma

In this study, we first performed a bioinformatics analysis and observed that high mPRα mRNA expression was associated with poor survival prognosis in patients with lung adenocarcinoma. Then, IHC was used to assess the expression of mPRα protein in lung adenocarcinoma tissues and to analyze its relationship with the prognosis of patients with lung adenocarcinoma. High protein expression levels of mPRα were also associated with poor patient prognosis. An mPRα knockdown lung adenocarcinoma cell line was constructed by transfecting cells with a lentiviral vector. The CCK‐8 and colony formation assay results showed that mPRα knockdown in lung adenocarcinoma cells could inhibit cell growth. Therefore, a comprehensive analysis of these results showed that mPRα is an important factor in promoting the development of lung adenocarcinoma.

Previous studies on the prognostic effects of progesterone‐related receptors in different cancer patients (such as breast, ovarian, and nonsmall cell lung cancer) focused on progesterone nuclear receptors^36^, ^37^, ^38^ but neglected hormone membrane‐associated receptors such as mPRα. Previously, for the first time, our group showed that mPRα is a potential prognostic indicator for breast cancer patients,^39^ and in this study, we showed that high mPRα expression is also a poor prognostic indicator for patients with lung adenocarcinoma. Thus, our results highlight the importance of mPRα in lung adenocarcinoma.

We observed that when mPRα was knocked down in lung adenocarcinoma cells, cell growth was significantly inhibited, indicating that the presence of mPRα maintains or promotes the growth of lung adenocarcinoma cells. However, as a membrane receptor protein, how does mPRα function in the absence of exogenous ligands? Perhaps the answer involves the activation mechanism of EGFR, another membrane receptor that is currently the most studied. In addition to its ability to bind to ligands to activate downstream pathways, EGFR can also self‐activate by forming dimers without ligand activation.^40^, ^41^ mPRα may also form dimers on the surface of cell membranes^42^, ^43^ or form membrane receptor complexes with other membrane‐associated proteins (such as PGRMC1),^44^ which are likely to be the mechanisms leading to mPRα self‐activation.

mPRα is also known as progestin and adipoQ receptor 7 (PAQR7). It has been reported that breast cancer cells can secrete adiponectin,^45^ indicating that lung adenocarcinoma cells may also have similar autocrine adiponectin functions, activating mPRα to exert a cancer‐promoting effect on lung adenocarcinoma. Previous studies showed that adiponectin can activate AMPK and its downstream effector mTOR in breast cancer through ERα, resulting in a stimulatory effect on the proliferation of breast cancer cells.^46^, ^47^ Therefore, we speculated that the mPRα‐mediated regulation of adiponectin may also influence the proliferation of lung adenocarcinoma through the AMPK/mTOR signaling pathway.

### P4 inhibits the growth of lung adenocarcinoma cells via mPRα

As discussed in the above section, mPRα is a receptor molecule that promotes the growth of lung adenocarcinoma cells. We hypothesized that mPRα may a potential drug target for the treatment of lung adenocarcinoma. Our team previously showed that P4 can play a role in inhibiting the invasion and migration of lung adenocarcinoma cells via mPRα.^18^ In this study, P4 and its derivative Org were used to treat lung adenocarcinoma cells. The CCK‐8 and colony formation assay results showed that both P4 and Org could inhibit the growth of lung adenocarcinoma cells through mPRα.

Since lung adenocarcinoma cells have both progesterone membrane receptor and progesterone nuclear receptor, when P4 is used to treat lung adenocarcinoma cells, it can bind to both these receptors. To confirm that the inhibitory effect of P4 on lung adenocarcinoma cell growth is mediated by mPRα, in addition to mPRα knockdown experiments in lung adenocarcinoma cells, cells were also treated with Org. Org is a derivative of P4 in which a methylene group has been added to the molecular structure of P4. A previous study showed that Org does not bind to PR, and because its affinity with mPRα is significantly better than that of P4, it is believed that Org is a specific agonist of mPRα.^24^ The cell function assay results from this study showed that P4 and Org have similar effects in inhibiting the growth of lung adenocarcinoma cells, and P4/Org failed to show significant inhibitory effects in mPRα knockdown lung adenocarcinoma cells. These results indicate that the anticancer effect exerted by P4/Org is mediated via mPRα.

mPRα is a G‐protein coupled receptor (GPCR) in which the intracellular carboxy terminus is coupled to a G protein.^10^ The G protein in the resting state is in a heterotrimeric state consisting of α, β and γ subunits. When the ligand binds to the GPCR, it activates the G protein, which separates the Gα subunit from the Gβγ subunit to exert its corresponding regulatory function.^48^ The Gα subunit is subdivided into Gαi and Gαs subtypes, in which Gαi is activated to inhibit the activity of adenylyl cyclase, thereby downregulating the level of intracellular cAMP.^48^ cAMP is a key regulator of energy metabolism in cells, and cAMP plays a major role in regulating the activity of PKA.^49^

The role of PKA is to phosphorylate downstream protein molecules that are involved in important signaling pathways, such as the PKA/CREB, PKA/β‐catenin and PKA/NF‐κB pathways.^30^, ^31^, ^32^, ^33^, ^34^, ^50^ The results of WB experiments in this study showed that P4/Org significantly inhibited the PKA/CREB and PKA/β‐catenin signaling pathways in lung adenocarcinoma PC‐9 cells but did not affect PKA/NF‐κB signaling pathway. Therefore, the specific mechanism of P4/Org inhibition of lung adenocarcinoma cell growth can be summarized as follows: P4/Org → binding mPRα→activation of Gαi → downregulation of cAMP→inhibition of PKA/CREB and PKA/β‐catenin signaling pathways→inhibition of lung adenocarcinoma cell growth.

PKA contains two regulatory and two catalytic subunits. In general, cAMP regulates PKA by first binding to the regulatory subunit of PKA to alter its conformation, causing the regulatory and catalytic subunits to dissociate, after which the catalytic subunits are released to perform their catalytic function.^51^ This entire process does not involve phosphorylation changes in PKA. The results of our current study showed that the mPRα‐mediated activity of P4/Org, not only affects the phosphorylation status of multiple pathway proteins downstream of PKA but also inhibits the phosphorylation level of PKA itself. As a protein kinase, PKA can autophosphorylate or be phosphorylated by other kinases,^52^, ^53^ suggesting that the mPRα‐mediated activity of P4/Org can also inhibit that of PKA through other mechanisms that require further study.

CREB is an important molecule that promotes the development of cancer, and inhibiting the activity of CREB in cancer cells is one of the goals of cancer treatment research.^54^, ^55^ However, there are currently no clinically applicable CREB inhibitors in the field of cancer research. The continued activation of the PKA/CREB pathway significantly promotes the growth of a variety of cancer cells, including lung cancer cells.^31^, ^56^ β‐catenin is normally regulated by the Wnt/β‐catenin pathway,^57^ but PKA/β‐catenin is also an important signaling pathway involved in cancer promotion.^32^, ^58^ The results of the current study showed that P4/Org can synergistically inhibit the above two pathways through mPRα, providing a new strategy for the treatment of lung adenocarcinoma.

The results of this study also showed that P4/Org treatment can also inhibit the PKA/CREB pathway in mPRα knockdown lung adenocarcinoma cells, suggesting that other receptors were involved in addition to the mPRα‐mediated effects in this process. These receptors that also exert P4/Org‐mediated effects may be additional progesterone membrane receptor subtypes. P4 binds to membrane receptors such as mPRδ and mPRε, which also function as GPCRs, and studies have shown that Org also has better binding affinity for mPRδ and mPRε.^8^ Although mPRδ and mPRε may mediate the effects of P4/Org on related pathways in lung adenocarcinoma cells, according to the cell function assay results in this study, they do not mediate the ability of P4/Org to exert significant tumor suppression effects.

Our nude mouse tumor‐bearing model results showed that the P4/Org treatment had a tendency to inhibit the growth of the lung adenocarcinoma model in vivo. However, only Org showed significant inhibition in the early stages of tumor model growth. When the growth time of the tumor model was prolonged, the inhibitory effect of P4/Org in the tumor model showed large individual differences, indicating that the tumor suppressing efficacy of P4/Org alone in the complex physiological/pathological conditions of the tumor model was limited. These results suggest that if P4/Org is further applied to the treatment of lung adenocarcinoma in the future, this treatment will need be given at an early stage, potentially together with other types of anticancer drugs.

In conclusion, our current study revealed the following. First, the high expression of mPRα is a potential poor prognosis marker in patients with lung adenocarcinoma. Second, mPRα can promote the development of lung adenocarcinoma and is a potential target for treatment, and finally that mPRα mediates the inhibitory effect of P4 on the PKA/CREB and PKA/β‐catenin signaling pathway to suppress the growth of lung adenocarcinoma cells.

## Disclosure

No authors report any conflict of interest.

## Supporting information


**Figure S1** Lentiviral vector GV248: hU6‐MCS‐Ubiquitin‐EGFP‐IRES‐puromycin
**Figure S2** The infection effects of lentivirus with MOI = 50/100/200 on lung adenocarcinoma A549 and PC‐9 cells.
**Figure S3** Lentiviruses were infected with A549 and PC‐9 cells with MOI = 100.
**Figure S4** The effect of lentivirus vector infection on the mRNA expression of mPRα in lung adenocarcinoma A549 and PC‐9 cells.
**Figure S5** The effect of lentivirus vector infection on the expression of mPRα protein in lung adenocarcinoma A549 and PC‐9 cells.
**Figure S6** The results of HE staining of liver and kidney in nude mice after 4 weeks of P4/Org intervention.
**Table S1** shRNA sequences.
