# Supplementary material for: Progesterone/Org inhibits lung adenocarcinoma cell growth via membrane progesterone receptor alpha
Source: Thorac Cancer. 2020 Jun 11;11(8):2209–23. doi: 10.1111/1759-7714.13528 (PMC7396388; doi:10.1111/1759-7714.13528)
Supplement: Supplementary file 1 — Figure S1 Lentiviral vector GV248: hU6‐MCS‐Ubiquitin‐EGFP‐IRES‐puromycin Figure S2 The infection effects of lentivirus with MOI = 50/100/200 on lung adenocarcinoma A549 and PC‐9 cells. Figure S3 Lentiviruses were infected with A549 and PC‐9 cells with MOI = 100. Figure S4 The effect of lentivirus vector infection on the mRNA expression of mPRα in lung adenocarcinoma A549 and PC‐9 cells. Figure S5 The effect of lentivirus vector infection on the expression of mPRα protein in lung adenocarcinoma A549 and PC‐9 cells. Figure S6 The results of HE staining of liver and kidney in nude mice after 4 weeks of P4/Org intervention. Table S1 shRNA sequences. [file TCA-11-2209-s001.docx]

**Progesterone/Org inhibits lung adenocarcinoma cell growth via**

**membrane progesterone receptor alpha**

**Supplementary Materials**

**Lentiviral vector related information**

The lentiviral vector used in this study to knock down the expression of PAQR7 (the gene that expresses mPRα) was constructed and packaged by Genechem (GeneChem Co. Ltd., Shanghai, China). We utilized six recombinant lentiviral vectors for PAQR7 knockdown screening. The details are as follows:


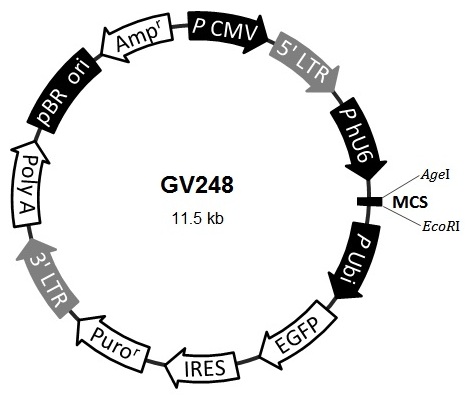


**Supplementary Figure S1.** Lentiviral vector GV248: hU6-MCS-Ubiquitin-EGFP-IRES-puromycin

**Supplementary Table S1.** shRNA sequences

| **Constructed lentiviral vector** |  | **Abbr.** | **5’** | **STEM** | **Loop** | **STEM** | **3’** |
| --- | --- | --- | --- | --- | --- | --- | --- |
| Lv-PAQR7-RNAi (44607-1) |  | Lv-44607 | Ccgg | gcCTTGGCACACTTCTACTAT | CTCGAG | ATAGTAGAAGTGTGCCAAGGC | TTTTTg |
| Lv-PAQR7-RNAi (44608-1) |  | Lv-44608 | Ccgg | ctGGCATTACAGCTTCTTCTT | CTCGAG | AAGAAGAAGCTGTAATGCCAG | TTTTTg |
| Lv-PAQR7-RNAi (44609-1) |  | Lv-44609 | Ccgg | ctTCTATTTCCGCACGCTGTT | CTCGAG | AACAGCGTGCGGAAATAGAAG | TTTTTg |
| Lv-PAQR7-RNAi (54304-1) |  | Lv-54304 | Ccgg | CAGCGCAAACTTGATCAGAAG | CTCGAG | CTTCTGATCAAGTTTGCGCTG | TTTTTg |
| Lv-PAQR7-RNAi (54305-1) |  | Lv-54305 | Ccgg | TATAACAAGTACATCCAGAAA | CTCGAG | TTTCTGGATGTACTTGTTATA | TTTTTg |
| Lv-PAQR7-RNAi (54306-1) |  | Lv-54306 | Ccgg | GCCGTACATCTATGCGGGCTA | CTCGAG | TAGCCCGCATAGATGTACGGC | TTTTTg |

Note: The insertion sequence of the lentiviral vector negative control (Lv-NC) was TTCTCCGAACGTGTCACGT

**Screening of lung adenocarcinoma cell lines down-regulated by mPRα**

We used six lentiviral vectors (Lv-44607, Lv-44608, Lv-44609, Lv-54304, Lv-54305 and Lv-54306) and two negative control vectors (Lv-NC1 and Lv-NC2) for screening. Lentiviruses infected lung adenocarcinoma A549 and PC-9 cells with MOI=50/100/200, respectively. The results showed that all different MOIs had good viral infection effect on the two lung adenocarcinoma cells (but the effect of MOI=100/200 was better than that of MOI=50, while the effect of MOI=100 was similar to that of MOI-200) (Supplementary Figure S2). Therefore, all the subsequent experiments were used MOI=100 to infect A549 and PC-9 cells (Supplementary Figure S3).


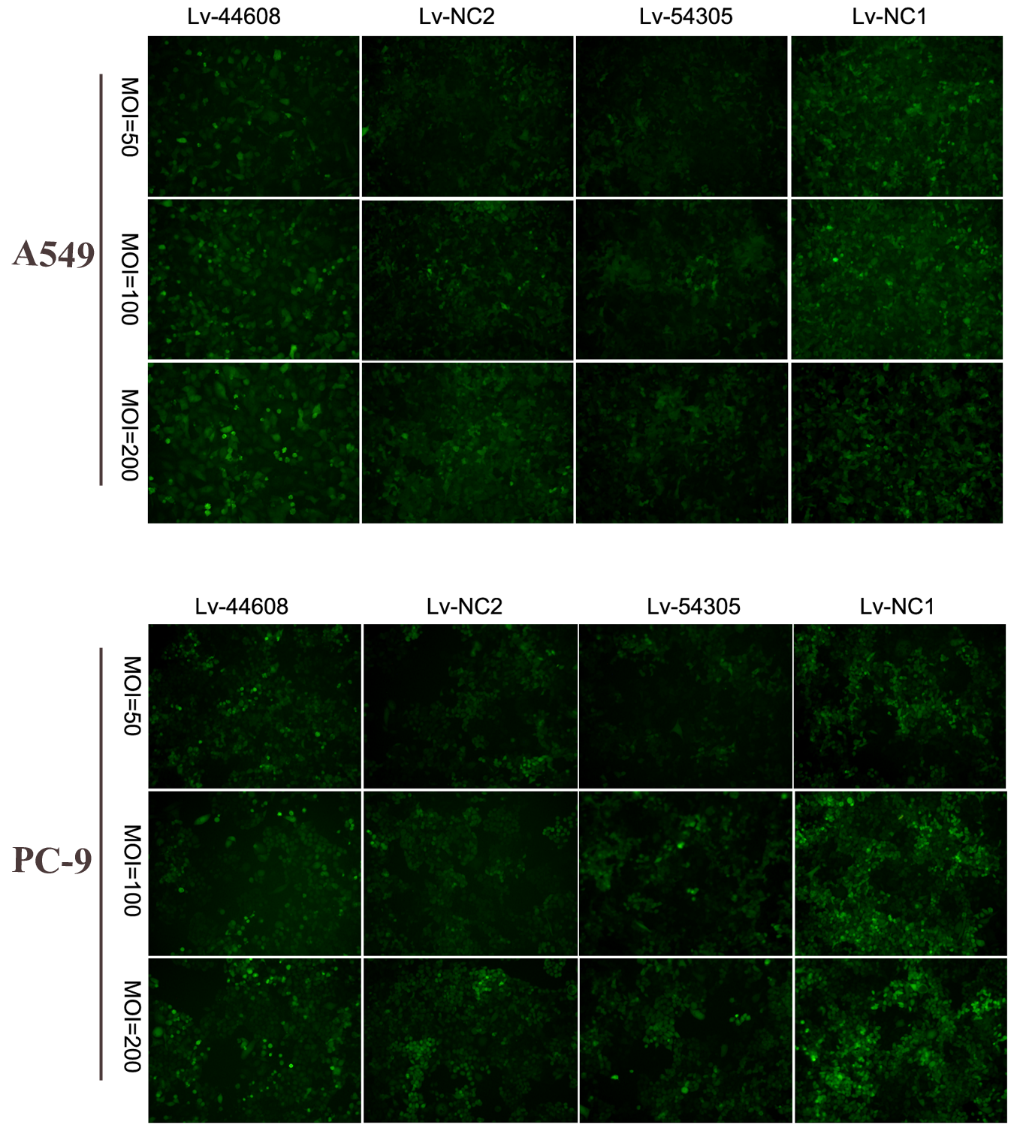


**Supplementary Figure S2.** The infection effects of lentivirus with MOI=50/100/200 on lung adenocarcinoma A549 and PC-9 cells.


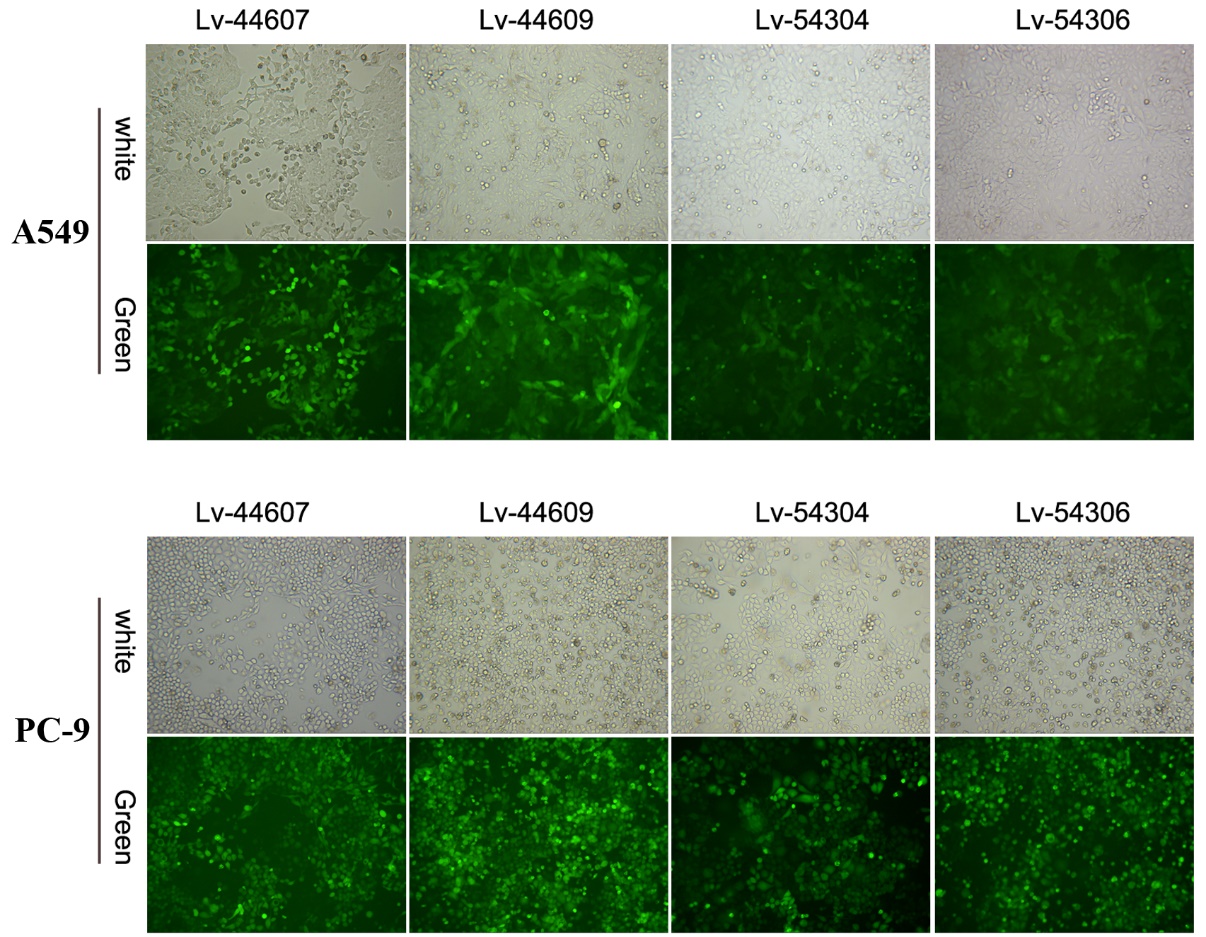


**Supplementary Figure S3.** Lentiviruses were infected with A549 and PC-9 cells with MOI=100.

qRT-PCR and WB experiments were used to detect the effects of lentiviral vector infection on the mRNA (Supplementary Figure S4) and protein (Supplementary Figures S4) expressions of mPRα in A549 and PC-9 cells. The results showed that Lv-44607 had a better knockdown effect on mPRa (both at the level of mRNA and protein) in both A549 and PC-9 cells compared with control group (no lentivirus vector was transfected) and Lv-NC group (negative lentivirus vector was transfected) (Supplementary Figures S4-5). However, the results also showed that Lv-NC had a significant knock-down effect on the expression of mPRα at protein level in A549 cells (Supplementary Figure S5). Therefore, PC-9 cells were selected for subsequent cell function experiments in this study.


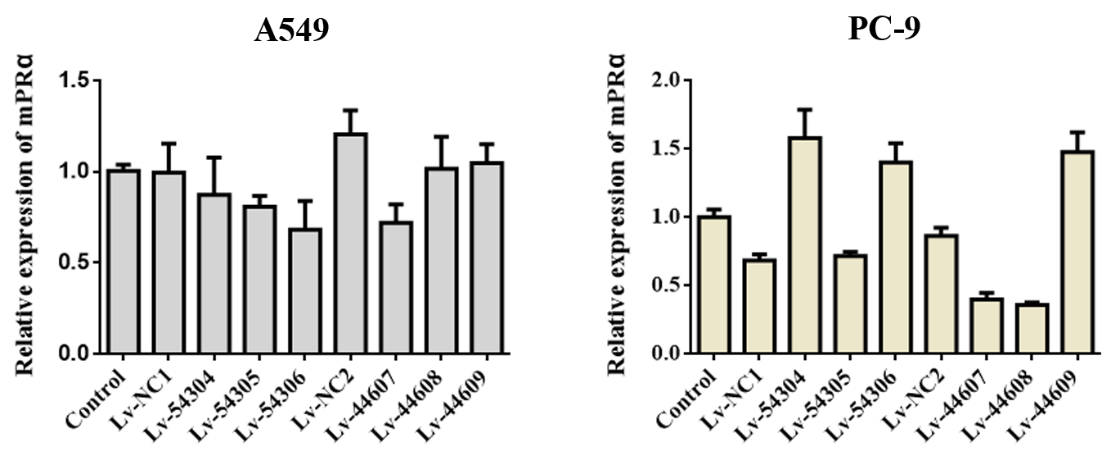


**Supplementary Figure S4.** The effect of lentivirus vector infection on the mRNA expression of mPRα in lung adenocarcinoma A549 and PC-9 cells.


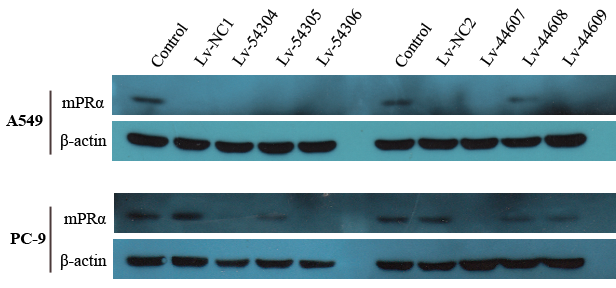


**Supplementary Figure S5.** The effect of lentivirus vector infection on the expression of mPRα protein in lung adenocarcinoma A549 and PC-9 cells.

**Effects of P4/Org intervention on liver and kidney tissues in nude mice**

HE staining showed that P4 or Org did not damage the liver and kidney of nude mice (Supplementary Figure S6).


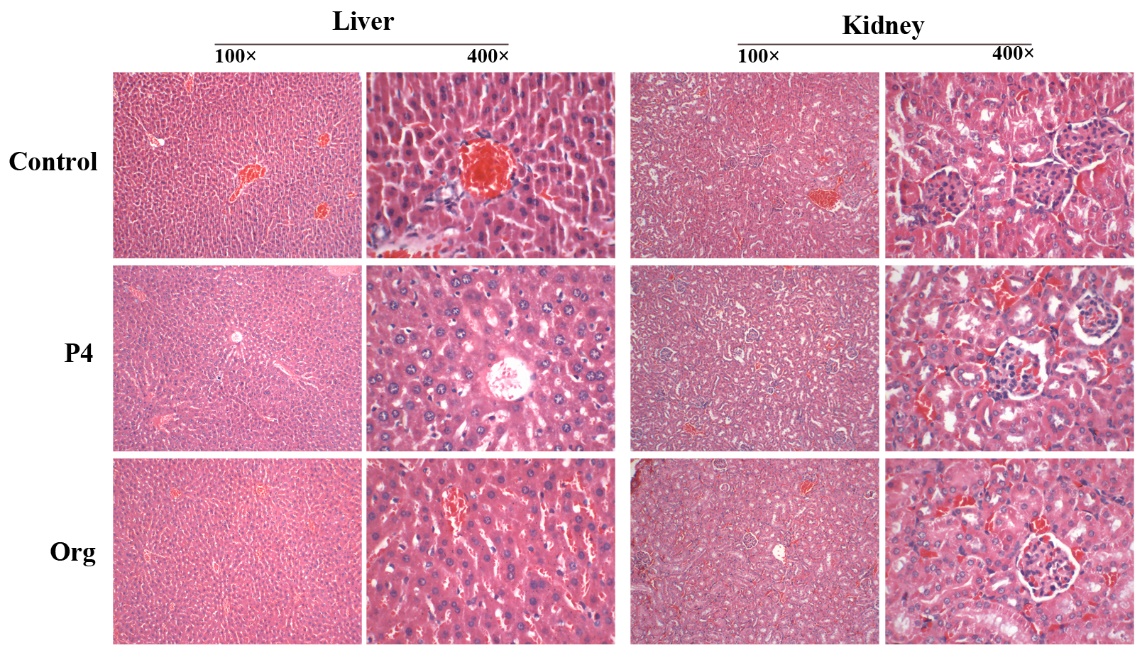


**Supplementary Figure S6.** The results of HE staining of liver and kidney in nude mice after 4 weeks of P4/Org intervention.
